# Analysis of ESTs from *Lutzomyia longipalpis* sand flies and their contribution toward understanding the insect–parasite relationship^☆^

**DOI:** 10.1016/j.ygeno.2006.06.011

**Published:** 2006-12

**Authors:** Rod J. Dillon, Al C. Ivens, Carol Churcher, Nancy Holroyd, Michael A. Quail, Matthew E. Rogers, M. Bento Soares, Maria F. Bonaldo, Thomas L. Casavant, Mike J. Lehane, Paul A. Bates

**Affiliations:** a Liverpool School of Tropical Medicine, Pembroke Place, Liverpool L3 5QA, UK; b The Sanger Institute, Wellcome Trust Genome Campus, Hinxton, Cambridge, UK; c Children's Memorial Research Center and Northwestern University, Chicago, IL 60611, USA; d Department of Electrical and Computer Engineering, University of Iowa, Iowa City, IA 52242, USA

**Keywords:** *Lutzomyia*, Expressed sequence tag, *Leishmania*, Genomics, Immunity, Parasite, Midgut

## Abstract

An expressed sequence tag library has been generated from a sand fly vector of visceral leishmaniasis, *Lutzomyia longipalpis.* A normalized cDNA library was constructed from whole adults and 16,608 clones were sequenced from both ends and assembled into 10,203 contigs and singlets. Of these 58% showed significant similarity to known genes from other organisms, < 4% were identical to described sand fly genes, and 42% had no match to any database sequence. Our analyses revealed putative proteins involved in the barrier function of the gut (peritrophins, microvillar proteins, glutamine synthase), digestive physiology (secreted and membrane-anchored hydrolytic enzymes), and the immune response (gram-negative binding proteins, thioester proteins, scavenger receptors, galectins, signaling pathway factors, caspases, serpins, and peroxidases). Sequence analysis of this transcriptome dataset has provided new insights into genes that might be associated with the response of the vector to the development of *Leishmania.*

The leishmaniases are a group of important neglected diseases with approximately 2,000,000 new cases every year and one-tenth of the world's population at risk of infection (www.who.int/tdr/diseases/leish). Symptoms of leishmaniasis range from relatively benign cutaneous disease through to potentially fatal visceral disease. The various parasites are all transmitted by certain species of female phlebotomine sand flies, and of these, *Lutzomyia longipalpis* is particularly significant, being the main vector of visceral leishmaniasis in South America [1]. The global risk of leishmaniasis is increasing, and the colonization of urban areas by *Lu. longipalpis* appears to be a significant factor in the recent increase in visceral leishmaniasis in South America [2]. Unfortunately, there are no vaccines or prophylactic drugs for leishmaniasis currently available, and chemotherapy is reliant on a small number of drugs. These factors indicate that control of the sand fly vector will remain an important component of leishmaniasis control for the foreseeable future [3].

Rearing the diminutive sand fly under laboratory conditions is a challenging process, and the limited amount of biological material that can be obtained from sand flies, for example, in comparison to mosquitoes and *Drosophila,* has been an obstacle to the study of responses to the *Leishmania* parasite. A major step forward will be to develop transcriptome information for the sand fly vector to accompany that now available for the parasites. For example, following the publication of the *Drosophila* and *Anopheles* mosquito genomes there has been rapid progress in the use of transcriptome information from these insects and the development of microarrays to study insect gut–microbe interactions (e.g., [4], [5]). In contrast, there have been remarkably few molecular studies of any kind examining sand fly genes that might influence *Leishmania* development. Those performed to date include a study of secreted salivary gland proteins [6], characterization of certain midgut digestive enzymes [7], [8], a differential expression study [9], characterization of a sand fly defensin [10], and the identification of a midgut epithelial galectin implicated in binding of the *Leishmania* parasite [11].

The gut of the hematophagous insect is a potentially nutrient-rich but highly specialized environmental niche, and the successful development of ingested potential pathogens or parasites such as *Leishmania* depends on their ability to avoid or adapt to the dramatic changes in the physicochemical environment accompanying blood-meal and sugar-meal digestion. The strategy of the mosquito-borne malaria parasite is to exit rapidly through the gut epithelium and continue development in the hemocoel. In contrast African trypanosomes in tsetse flies [12] and *Leishmania* [13] have adapted to remaining and developing in the insect gut. The *Leishmania* parasite is supremely adapted to the gut environment of the sand fly, secreting a unique gel-like material composed mainly of a high-molecular-weight filamentous proteophosphoglycan (fPPG [14]). *Leishmania* fPPG serves a dual function, first blocking the fly gut and improving chances for transmission and subsequently aiding survival of the parasite in the mammalian host [15], [16]. Although the parasites are confined to the gut lumen, *Leishmania* is expected to have a wider impact on gene regulation in other tissues such as the fat body and ovaries. Therefore, a whole-body-derived cDNA library was generated in the current study.

Interpretation of the resulting data is helped by the order Diptera containing the two best studied insect genomes, *Anopheles gambiae* and *Drosophila melanogaster,* and information on two other hematophagous Diptera has also recently become available: the tsetse fly *Glossina morsitans* (http://www.sanger.ac.uk/Projects/G_morsitans/) [17], and the mosquito *Aedes aegypti* (http://www.tigr.org/msc/aedes/aedes.shtml) [18]. For comparative purposes, it should be noted that phlebotomine sand flies are more closely related to *Anopheles* and *Aedes,* belonging to the dipteran suborder Nematocera along with many bloodsucking insects (mosquitoes, blackflies, and biting “midges”), whereas *Glossina* and *Drosophila* are found in the other suborder (Brachycera). The availability of these dipteran genome resources has facilitated the sequence identification and annotation of the *Lutzomyia* data described here and online (http://www.sanger.ac.uk/Projects/L_longipalpis/). This transcriptome study will provide the platform for the development of microarrays and will provide further impetus to identifying the insect genes involved in regulating *Leishmania* development in the vector.

## Results and discussion

A total of 16,608 cDNA clones from a normalized library of female *Lu. longipalpis* were sequenced from both ends*.* A total of 33,216 reads were attempted and of these 26,495 were successful (80% pass rate). An additional 1728 reads were attempted from the unnormalized cDNA library, with 1433 obtained. The assembly with Phrap [19] generated 5210 contigs (mean length, 1225 bp) and 4993 singlets (mean length, 605 bp), giving a total of 10,203 ESTs. The average number of reads per contig was 4.4. Most of the sequences were novel sand fly sequences; a comparison of assembled contigs with the 1309 *Lutzomyia* spp. (highly redundant) DNA sequences available in the public databases (August 2005) revealed only 222 similar sequences occurring among the contigs with an E value of less than 10^−25^. Comparison with 379 *Phlebotomus* spp. DNA sequences revealed 160 hits with the same E value.

The sequences were compared using Blastx to the UniProt database to identify the number of transcripts without a significant match and thereby obtain an estimate of putative novel genes (Supplementary Table S1). A total of 5962 (58.4%) sequences had matches at the *E* = 10^− 5^ cutoff, i.e., were similar to known genes; 1624 had no hits and 2617 failed to meet the E value threshold; thus up to 4241 (41.6%) of the sequences may be novel. Comparison with *Drosophila* and *Anopheles* databases gave similar results, with estimates of sequences possessing no similarity of 44.7 and 45.9%, respectively.

**Supplementary Fig. S1:**
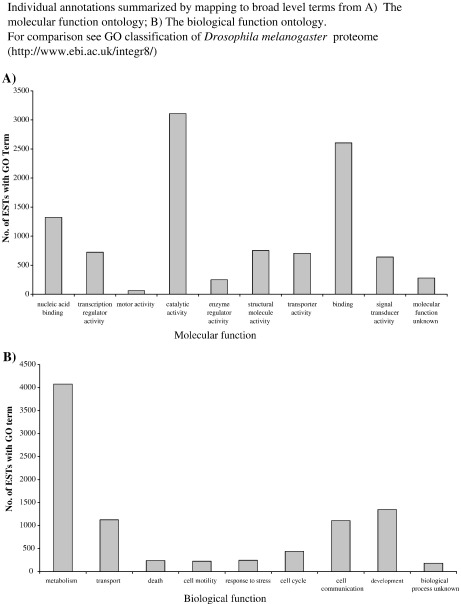
Classification of EST clusters into the main Gene Ontology functional classes.

The sand fly expressed sequence tags (ESTs) were categorized by selected GO terms (Supplementary Fig. S1). The proportions of GO terms are similar to those found in the *Drosophila* proteome analysis (http://www.ebi.ac.uk/integr8/). A total of 6460 (63.3%) sequences were assigned a putative molecular function term by transitive annotation of GO terms. It was not possible to give an accurate estimate of the proportion of the *Lutzomyia* transcriptome sequenced in the EST study. Some transcripts, for example, may represent nonoverlapping parts of the same gene and the annotation was largely automated. However, the current release of Ensembl (version 36—December 2005) lists over 14,300 genes in the *An. gambiae* genome and 5517 of our sequences had matches with *Anopheles* proteins; therefore, considering the library was derived solely from adult female sand flies it was apparent that a large proportion of the predicted *Lutzomyia* genes were represented in this study.

The cDNA was synthesized from a pool of RNA extracted from whole bodies of sand flies, some of which were infected with *Leishmania infantum, Le. mexicana,* or bacteria. The rationale was to produce a wide range of cDNAs that could be used to construct a cDNA microarray to explore gene expression throughout the whole insect in response to *Leishmania* or microbial infections. *Lu. longipalpis* is a permissive vector allowing the development of *Le. mexicana* as well as the naturally occurring species *Le. infantum;* thus including *Le. mexicana*-infected insects will allow comparisons with different parasite infections. Further, stimulation of antibacterial responses and inclusion of these cDNAs will enable us to dissect the roles of common or different immune pathways that sand flies may use in response to protozoan or bacterial infection. A midgut library would also be relatively limited in its utility, e.g., fat-body-related immune gene expression would be excluded. However, because infected flies were used the potential for microbial contamination of the library was a point of concern. It was expected that contamination with bacterial cDNA would be minimal, as their mRNAs lack poly(A) tails and would not be efficiently reverse transcribed. There were only 10 hits at an E value of less than 10^−20^ toward bacterial sequences in the cDNA library as determined using Blastn (Supplementary Table S2). Sand flies infected with *Leishmania* can contain 1–5 × 10^4^ parasites per gut (∼ 5–25 ng total RNA), which may represent 0.5–3.5% of the total RNA isolated from such flies. *Leishmania*-infected flies comprised ∼ 25% of the pool used for library construction. Therefore, it was anticipated that *Leishmania* nuclear-derived cDNA would contaminate the library by approximately 0.2–0.9% assuming no difference in efficiency of cDNA synthesis. Blastn was used to screen the library against *Leishmania major* (the most mature of the three *Leishmania* genome databases available), and 25 (0.24%) contigs or singlets had hits with E values less than 10^−25^, i.e., constituted likely *Leishmania* genes (Supplementary Table S3); this value is at the lower end of the expected range of contamination. Most of these putative *Leishmania* genes are highly conserved and fulfill a housekeeping function, although there is a putative lipase (Supplementary Table S3; NSFM-46a03.p1k) that is clearly a *Leishmania*-derived sequence.

It should be noted that the cDNA library construction involved an essential size selection step, whereby cDNA of < 350 bp was excluded. This was necessary to prevent the library from being overwhelmed by short sequences. The drawback of the approach was that the library was unlikely to contain transcripts for small genes such as those encoding some of the immune peptides.

The EST sequences were examined for potential genes of interest with regard to *Leishmania*–sand fly interactions. Since *Leishmania* development is confined to the insect gut, this analysis focussed on elements that might be involved in the structural composition of the gut particularly related to the epithelial barrier, the peritrophic matrix barrier, the insect immune response, and genes related to digestion of the blood and sugar meals. All the contigs described in the following text are listed in Table 1. Further alignment information is also given in supplementary figures. A number of ESTs with complete homology to previously described *Lu. longipalpis* salivary gland genes were present; these are not described here and the interested reader should consult Ref. [6].

**Table 1:** Summary of selected *Lu. longipalpis* ESTs and their putative functions

	Contig	Length (bp)	Putative function	Score	E value	Homology	
*Structural*							
1	NSFM-42c02.q1k	1755	Glutamine synthetase	1562	1.10E–160	AF395490	Aa
2	NSFM-51c10.p1k	1511	Glutamine synthetase	1225	6.60E–125	AF395490	Aa
3	NSFM-43h06.p1k	1301	Mucin-like	165	3.00E–39	NP-002448	Hs
4	NSFM-144a01.q1k	1660	Mucin-like	225	4.00E–57	NP-002448	Hs
5	NSFM-161h05.q1k	1311	Peritrophin-like	110	1.00E–22	AAN63949	Px
6	NSFM-72d06.q1k	1222	Peritrophin-like	1175	3.00E–42	AAM21354	Cf
7	NSFM-114b12.p1k	966*	Peritrophin-like	96	1.00E–18	CG31077	Dm
8	NSFM-129g05.q1k	420	Hemomucin	174	8E–43	AAC47118	Dm
9	NSFM-129g05.p1k	559	Hemomucin				
10	NSFM-126e12.q1k	795*	Microvillar protein/insect allergen	156	8.00E–37	AY050565	Aa
11	SFM-02f01.p1ka	784*	Microvillar protein/insect allergen	96	1.00E–18	AY050565	Aa
12	NSFM-19a10.p1k	761*	Microvillar protein/insect allergen	140	4.00E–42	AY050565	Aa
13	NSFM-154e02.p1k	731*	Microvillar protein/insect allergen	167	3.00E–40	AY050565	Aa
14	NSFM-40c09.p1k	1053	β-integrin	444	1.00E–123	AJ292755	Ag

*Immunity*							
15	NSFM-148h05.p1k	1158*	Galectin A	392	1.00E–108	AY538600	Pp
16	NSFM-165c04.q1k	1366*	Galectin B	307	4.00E–82	ENSANGP00000014884	Ag
17	NSFM-154d08.q1k	1640	Galectin C	302	1.00E–80	ENSANGP00000016692	Ag
18	NSFM-47b01.p1k	1182	Galectin D	157	6.00E–37	ENSANGP00000025712	Ag
19	NSFM-165h06.q1k	2010	Leucine-rich repeats	113	2.00E–23	ENSANGP00000006849	Ag
20	NSFM-165a10.q1k	1217	Leucine-rich repeats	108	3.00E–22	ENSANGP00000014441	Ag
21	NSFM-79f04.p1k	1272	TEP15	404	1.00E–111	Q7Q4E8	Ag
22	NSFM-14b06.p1k	1309*	GNBP A	91	6.00E–17	GA18590	Dp
23	NSFM-111b04.p1k	903*	GNBP A	217	3.00E–55	ENSANGP00000020260	Ag
24	NSFM-140g04.q1k	1175*	GNBP B3	521	1.00E–146	ENSANGP00000017035	Ag
25	NSFM-81b08.p1k	1256	Peptidoglycan recognition PGRP-LB	244	4.00E–63	CG14704	Dm
26	NSFM-04c02.p1k	973	Peptidoglycan recognition PRGP-LC-like	228	3.00E–58	ENSANGP00000029037	Ag
27	NSFM-40a05.q1k	1405	SCR class B	352	1.00E–95	ENSANGP00000012643	Ag
28	NSFM-123e08.q1k	766	SCR class B	105	1.00E–21	ENSANGP00000012652	Ag
29	NSFM-84c11.q1k	512	SCR class B	215	3.00E–55	ENSANGP00000012656	Ag
30	NSFM-154g05.p1k	1534	SCR class C	255	3.00E–66	ENSANGP00000015204	Ag
31	NSFM-73e11.q1k	1473*	Serpin	308	2.00E–82	ENSANGP00000015833	Ag
32	NSFM-01f02.p1k	1458*	Serpin	107	1.00E–21	CG9460	Dm
33	NSFM-37b05.p1k	1322*	Caspase-7	272	2.00E–71	AAO92598	As
34	NSFM-156e03.p1k	1505	Spaetzle	108	5.00E–22	CG6134	Dm
35	NSFM-165a11.q1k	2027*	Cactus	296	2.00E–80	CG5848	Dm
36	NSFM-109f03.q1k	1191	Tube	95	7.00E–20	CG10520	Dm
37	NSFM-162d06.p1k	1265*	Serine protease Easter precursor	260	6.00E–68	P13582	Dm
38	NSFM-30c10.p1k	1507*	TNFR superfamily protein Wengen	99.8	2.00E–19	CG6531	Dm
39	NSFM-119e05.q1k	840	Thioredoxin reductase	728	9.50E–73	CAD30858	Ag
40	NSFM-15h07.q1k	832	Thioredoxin reductase	1053	3.50E–107	CAD30858	Ag
41	NSFM-106g11.q1k	890*	Thioredoxin peroxidase (peroxiredoxin)	308	1.00E–82	Q8WSF6	Aa
42	NSFM-21g01.p1k	898*	Thioredoxin peroxidase (peroxiredoxin)	749	5.30E–75	Q8WSF6	Aa
43	NSFM-03h04.p1ka	874*	Thioredoxin peroxidase (peroxiredoxin)	752	2.60E–75	Q8WSF6	Aa
44	NSFM-18g09.p1k	852	Thioredoxin peroxidase (peroxiredoxin)	320	8.00E–88	CG5826	Dm
45	NSFM-38g02.q1k	1044*	Thioredoxin 1	156	1.00E–36	AF236124	Ag
46	NSFM-39d09.q1k	873*	Superoxide dismutase	216	5.00E–55	ENSANGP00000015824	Ag
47	SFM-05g07.q1ka	1591*	Peroxidase	264	4.00E–63	ENSANGP00000019589	Ag
	SFM-05g07.q1ka		Catalase	196	2.00E–50	CG8913	Dm
48	NSFM-83c09.p1k	1272	Peroxidase	182	1.00E–44	ENSANGP00000019589	Ag
	NSFM-83c09.p1k		Catalase	145	6.00E–35	CG8913	Dm
49	NSFM-156c09.p1k	1590	Xanthine dehydrogenase	720	0.00E+00	ENSANGP00000025172	Ag
50	NSFM-106d08.q1k	1242*	Glucuronosyltransferase activity	395	2.00E–108	ENSANGP00000020582	Ag
51	NSFM-123b01.p1k	773*	Lysozyme i-1	176	5.00E–43	AAT51799	Ag
52	NSFM-94f03.q1k	2215	Calreticulin	296	2.00E–78	BAB79277	Gm
	NSFM-94f03.q1k		Calreticulin	293	2.00E–77	AAL68781	Ag
53	NSFM-153c02.p1k	1289	Transferrin	316	1.00E–84	AAL58077	Aa
54	NSFM-152e11.q1k	1505*	Zinc/iron transporter Zip3	238	7.00E–63	CG6898	Dm
55	NSFM-144g07.q1k	1104	Ferritin	147	4.00E–34	AAL47694	Gm
56	NSFM-71d08.p1k	1215*	p38 MAP kinase	602	7E–171	BAE46743	Bm
57	NSFM-134g07.q1k	1161	CDK5 kinase	518	9E–146	CAA67861	Dm

*Digestion*							
58	NSFM-165c07.q1k	845*	Trypsin	374	2.00E–102	AAM96943	Pp
59	NSFM-94b08.q1k	941*	Trypsin	322	1.00E–86	AAM96942	Pp
60	NSFM-02a01.p1ka	891*	Trypsin	291	1.00E–77	AAM96940	Pp
61	NSFM-01d03.q1k	949*	Chymotrypsin	344	3.00E–93	AAM96939	Pp
62	NSFM-36e10.q1k	880*	Chymotrypsin	337	2.00E–91	AAM96939	Pp
63	NSFM-27h12.q1k	908*	Chymotrypsin	300	5.00E–80	AAM96939	Pp
64	NSFM-119c09.p1k	1463	Aminopeptidase N	236	1.00E–60	AAK73351	Aa
65	NSFM-28e11.p1k	1420*	Aminopeptidase N puromycin sensitive	257	8.00E–67	ENSANGP00000004374	Ag
66	NSFM-74b03.q1k	1768	Aminopeptidase N	553	5.00E–156	XP-396261	Am
67	NSFM-03g02.p1ka	2223	Aminopeptidase A	762	0.00E+00	CG32473	Dm
68	NSFM-58d09.p1k	1869	Aminopeptidase, leucyl	704	0.00E+00	ENSANGP00000010351	Ag
69	SFM-05c11.q1k	1361*	Carboxypeptidase A	443	7.00E–123	AAD47827	Aa
70	NSFM-129d03.q1k	1490	α-glucosidase	473	1.00E–131	ENSANGP00000010269	Ag
71	NSFM-159e06.p1k	1389	β-glucosidase	535	2.00E–150	ENSANGP00000006376	Ag
72	NSFM-14f03.p1k	1245	Amylase	501	3.00E–140	CAA59126	Dm
73	NSFM-97c03.q1k	1705	Amylase	457	5.00E–127	CAA60857	Ag
74	NSFM-88d12.p1k	1038	Chitinase	517	3.00E–145	T14075	Aa
75	NSFM-18f06.q1k	1498*	Bacteria-responsive protein	528	2.00E–148	AAS80138	Ag
76	NSFM-126b06.q1k	1131*	Lipase	513	6.00E–144	AAO22149	Pp
77	NSFM-79b09.q1k	944*	SDR short chain reductase	384	2.00E–105	RH24570	Dm
78	NSFM-87c01.q1k	1159*	SDR short chain reductase	339	1.00E–91	ENSANGP00000021339	Ag
79	NSFM-15g12.p1k	1303*	Aquaporin	343	9.00E–93	CG12251	Dm
80	NSFM-83c08.p1k	746*	Aquaporin	248	1E–64	AAF64037	Aa

The contig (NSFM or SFM) is given for each gene. A “q” following the clone identifier indicates that sequencing was from the 3′ end of the clone. Asterisk next to length (bp) indicates presence of full sequence for putative protein. The lowest BLASTX *E* value (most significant similarity) together with putative function based on this homology is given. Ag, *Anopheles gambiae;* As, *Anopheles stephensi;* Aa, *Aedes aegypti;* Am, *Apis mellifera;* Bm, *Bombyx mori;* Cf, *Ctenocephalides felis;* Dm, *Drosophila melanogaster;* Dp, *Drosophila pseudoobscura;* Gm, *Glossina morsitans morsitans;* Hs, *Homo sapiens;* Pp, *Phlebotomus papatasi;* Px, *Plutella xylostella*.

### Structural proteins and the gut

The *Leishmania* parasite lives in close proximity to the gut epithelium and the peritrophic matrix (PM) during its insect developmental phase. The PM, which completely surrounds the blood meal, is secreted by the midgut epithelium within the first few hours after ingestion of the blood. Consequently, one critical point in the survival and development of the parasite is their escape through the PM. One reason for the loss of parasite infection in an unsuitable vector can be the failure of the parasite to escape from the PM before it is voided from the gut with the digested remnants of the blood meal [20]. Glutamine synthetase (EC 6.3.1.2) is a key enzyme involved in the biosynthesis of chitin and the PM [21]. Two orthologs of the *Ae. aegypti* enzyme (Table 1, contigs 1 and 2) that may be involved in the synthesis of the *Lu. longipalpis* PM were detected. Mucins and peritrophins are also major protein components of the PM [22]. In vertebrates, mucins are components of the intestinal mucous layer that protects the underlying epithelium and, therefore, fulfill a function analogous to the PM proteins. Several EST sequences with similarity to the threonine/serine-rich domains of human mucin-2 were found (contigs 3 and 4). The translated regions of these ESTs are potentially heavily O-glycosylated as predicted by NetOGlyc 3.1. Other putative O-glycosylated translated products with similarity to peritrophins were also present (contigs 5–7). One of these possessed a signal peptide and was translated to give a glycosylated sequence with similarity to cat flea peritrophin (contig 6) [23]. A related mucin-like partial sequence with similarity to *Drosophila* hemomucin [24] was also found, with a predicted signal peptide and transmembrane helix. Hemomucin is thought to be involved in the induction of antibacterial effector molecules [24]. The NSFM-129g05.q1k contig (No. 8) did not contain any glycosylation sites but its counterpart (No. 9) contained multiple O-glycosylation sites that in *Drosophila* act as ligands for a snail lectin (*Helix pomatia* A hemagglutinin).

Potential structural elements of the midgut epithelium were also identified. A number of contigs with homology to the *Ae. aegypti* microvillar membrane protein AEG12 were discovered (Table 1, contigs 10–13; Fig. S3) [25] and they also appear to be more distantly related to insect allergens, although sequence conservation is poor. AEG12 is an adult female-specific gene induced by blood-meal digestion, and interestingly the gene was significantly up-regulated in *Plasmodium gallinaceum*-infected *Ae. aegypti* females at 12 h postinfection [26]. The ORFs of the putative *Lu. longipalpis* proteins, which possess no more than 44% similarity with AEG12, all possess a signal peptide and a potential transmembrane domain. Also in common with AEG12 they possess two insect allergen-related repeat domains; however, unlike AEG12 they lack both GPI anchor and potential O-linked glycosylation sites. A putative homolog of an *An. gambiae* β-integrin [27] was detected (No. 14). The mosquito protein is expressed in the gut and regulated in response to blood feeding, with a peak in transcript abundance 48 h postfeeding. Integrins are also known to play a role in the interaction of microbes with host cells and have been implicated in the binding of *Leishmania* to human macrophages [28]. Although integrin has not been found on the microvillar surface of insects a putative role for integrin was suggested during the invasion of *Plasmodium* ookinetes [29]; the coating of ookinetes may occur after the parasite has come in contact with the inside of gut epithelial cells or after cell death and integrin release.

### Immune genes and the gut barrier

Immune reactions in insects are initiated by the recognition of conserved pathogen-associated molecular patterns (PAMPs) by corresponding insect pattern recognition receptors or PRRs. The latter may be cell surface bound or circulating in the hemolymph. Toll-like receptors (PRRs), notably TLR4, have been located in mammalian intestinal epithelia [30]. Several classes of potential PRRs have been detected in the EST library and are described below, including GALEs, TEPs, GNBPs, PGRPs, and SCRs.

**Supplementary Fig. S2:**
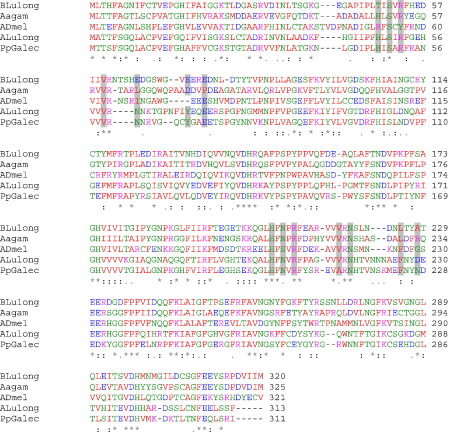
Alignment of putative *Lu. longipalpis* galectins A and B with the tandem repeat *P. papatasi* galectin (AY538600), *An. gambiae* Gale 2 (EAA06231) and *D. melanogaster* galectin (NP611349). Residues associated with B-galactoside binding are boxed in grey.

**Supplementary Fig. S3:**
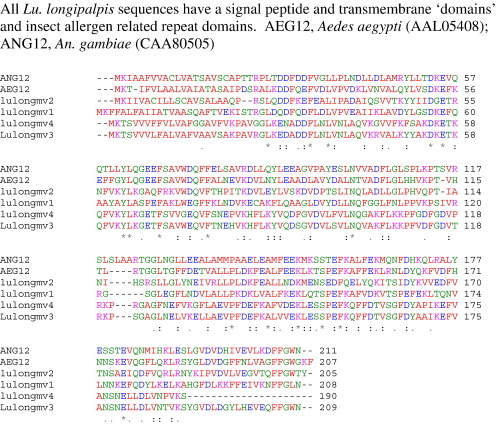
Alignment of putative secretory proteins with similarity to *Aedes* microvillar protein AEG12 and a related *Anopheles* protein.

Binding of *Leishmania* parasites to microvilli on the surface of the midgut epithelium is an important feature of successful parasite development in the sand fly [13]. Insect galectins are thought to function as PRRs, and a tandem repeat galectin (PpGalec) is expressed in the midgut of the sand fly *Phlebotomus papatasi* [11]. PpGalec was implicated in the specific binding of the *Le. major* parasite surface PAMP lipophosphoglycan (LPG), which possesses exposed Gal(β1–3) moieties on side-chain branches. These LPG side-chain sugars are involved in the species-specific attachment of *Leishmania* parasites in certain vectors, and the presence of PpGalec underpins the hypothesis of a GALE binding site. Interestingly, GALEs have also been described in other insects; for example, IGALE 20 (GALE 8) was up-regulated in *An. gambiae* in response to *Plasmodium* infection [31]. Four putative galectin-like proteins were identified by sequence homology in the present study (Nos. 15–18; Fig. S2). LulongGale A is a tandem repeat GALE with high similarity to PpGalec, which thus appears to fulfill a conserved function in sand flies. Interestingly, the *Leishmania* species transmitted by *Lu. longipalpis, Le. infantum,* does not bear galactose residues on its surface LPG [32], implying that a different receptor–ligand pair is responsible for midgut binding in this parasite–vector combination. Also, Western ligand blotting of microvillar extracts from *Ph. papatasi* midguts with *Leishmania* LPG revealed a number of LPG-binding proteins [33], and there are a number of other potential binding proteins that might be expressed on the microvillar surface. No sequences with homology to *An. gambiae* LRIM1 [34] were found in the present study, but there were a number of putative proteins with leucine-rich repeat motifs (Nos. 19 and 20), and proteins with such domains are thought to be involved in the initiation of the insect immune response involving Toll-like receptors. No ESTs with high similarity to C-type lectins or fibrinogen-related proteins were detected.

TEPs are endoproteinase inhibitors with an α_2_-macroglobulin domain, and a family of 15 TEPs was identified in the *An. gambiae* genome [35]. Of these TEP1 was found to bind to the surface of *Plasmodium berghei* ookinetes, leading to killing of the parasite [36]. Whether a similar family exists in *Lu. longipalpis* is unknown at present, but one putative TEP (contig 21) with high similarity to *An. gambiae* TEP15 was detected.

**Supplementary Fig. S4:**
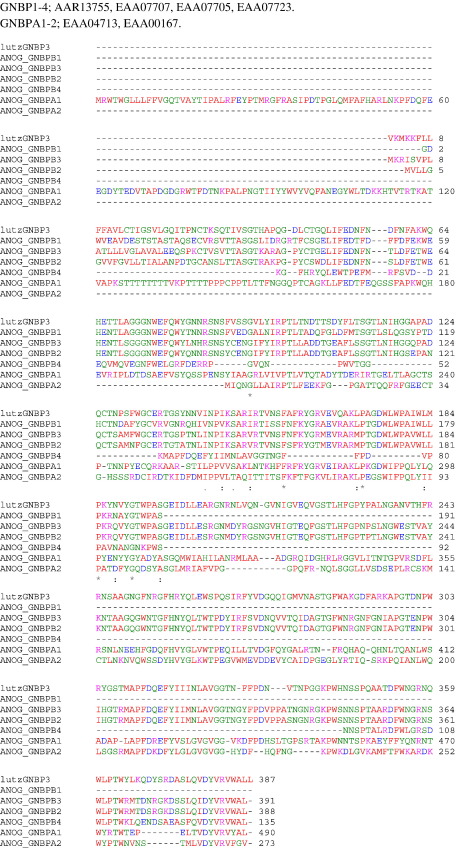
Putative *Lu. longipalpis* GNBP sequence alignment with *An. gambiae* GNBPB and GNPBA gene family.

Gram-negative binding proteins, as their name suggests, are induced mainly by bacterial infection. Six GNBPs were found in the *An. gambiae* genome, and GNBP A1 and A2 are the most closely related to *Drosophila* genes [35]. GNBP A1 is up-regulated during malarial (but not bacterial) infection of *An. gambiae* [35]. Interestingly, the GNBP A1-like gene CG12780 was also selectively up-regulated by oral protozoan infection but not by bacteria in *D. melanogaster* [4]. Two GNBP A1-like coding sequences (contigs 22 and 23) were found in *Lu. longipalpis,* with one (No. 22, E value 8.0E−06) possessing similarity to CG12780. Another GNBP, B1, is induced in the midgut and salivary glands of *An. gambiae* upon *Plasmodium* infection [5]. Homologs were not found in *Drosophila* and, therefore, it was postulated that the distinct GNBP B cluster had arose from a recent gene expansion in *Anopheles* [35]. However, the detection of contig 24 in *Lu. longipalpis,* which is more closely aligned to sequences in the subgroup B cluster than in the A cluster, indicates that the gene expansion relates to the Nematocera rather than specifically to *Anopheles* spp. (Fig. S4). Two other PRRs of relevance to bacterial infection were discovered in the *Lu. longipalpis* EST dataset (Nos. 25 and 26). These are orthologs of the peptidoglycan receptors PGRP-LB and PGRP-LC.

The final class of PRRs found were members of the scavenger receptor family. These are involved in recognition and phagocytosis, and there are three distinct classes in mosquitoes [35]. Three SCR class B-like proteins with putative CD36 domains were found in *Lu. longipalpis* (contigs 27–29). One of these (No. 28) is homologous with *D. melanogaster* CG2736, which was up-regulated during oral infection by a protozoan (*Octosporea* sp.), but not by bacteria [4]. Another sand fly contig was found (No. 30) with highest homology to the third class of *Anopheles* SCRs, which has only one member, and also to a protein previously reported in *Drosophila* (dSR-C). The Pfam analysis of the putative translated SCR revealed two sushi (complement control protein modules) domains, a MAM domain thought to have an adhesive function, and a somatomedin-B-like domain.

A putative protein with a signal peptide, similar to calreticulin, was identified (contig 52); it is involved in nonself recognition in invertebrate cellular defense reactions in *Galleria mellonella* [37], and a similar calreticulin was located in the salivary gland of *An. gambiae*[38].

**Supplementary Fig. S5:**
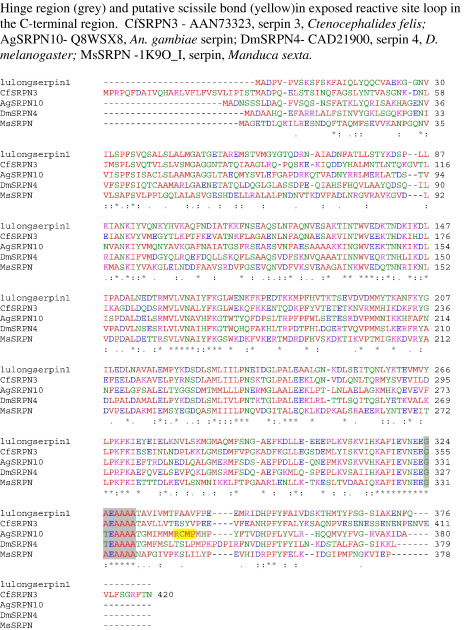
Alignment of putative serpin.

**Supplementary Fig. S6:**
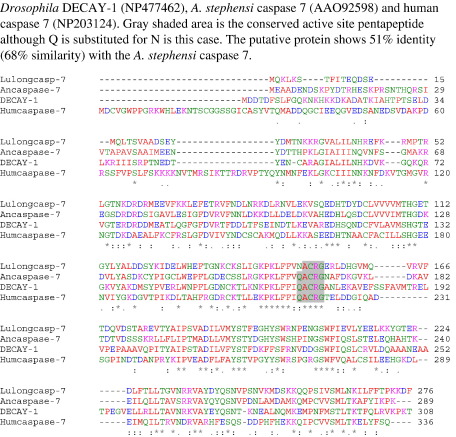
ClustalW alignment of deduced amino acid sequence of *Lu. longipalpis* caspase.

The regulation of the immune response in the gut epithelia is likely to be a key factor modulating the success of *Leishmania* development in the gut. Various immune-regulatory modulators have been described in insects. Serpins are modulators of the immune response acting via proteolytic cascades, and a number of putative serpins were found in *Lu. longipalpis* (contigs 31 and 32). One of these (No. 31) is very similar to the SRPN10 isoforms of *An. gambiae* that are potential markers for midgut invasion, as they are induced by *Pl. berghei* infection [39] (see Fig. S5). Invaded midgut cells undergo apoptosis, suggesting a link between this type of intracellular serpin and epithelial damage [40]. The sand fly serpin homolog lacks a signal peptide as do the SRPN10 isoforms. Although *Leishmania* parasites generally do not penetrate the gut epithelia, degenerated and/or damaged epithelial cells have been observed in the *Leishmania*-infected midgut (R.J. Dillon, unpublished observations). Apoptosis systems are involved in disposal of unwanted cells during development but also have potential roles in immunity; an effector caspase is induced and activated during ookinete invasion of *An. stephensi* [41]. An open reading frame with the complete coding region for a protein with homology to *An. stephensi* caspase-7 without a signal peptide was identified in *Lu. longipalpis* (No. 33; Fig. S6).

The most intensively studied aspect of the insect immune response is the regulation and action of AMPs. Toll and Imd pathways are implicated in AMP expression but it has been suggested that the induction of AMPs in epithelia is under the unique control of the Imd pathway in *Drosophil*a*,* as the epithelial response is compromised only in Imd mutants [42]. However, until recently [43] most experiments on insect immune regulation have used the “injection route” of microbes or their PAMPs into the hemolymph as the model rather than natural infection. Additionally, interpretation of these results is hampered by investigators using unsuitable species, unrealistic numbers of microbes, and heat treatments, which may disrupt antigen presentation.

De Gregorio suggested that the immune-regulated catalase (see below) is also controlled via the Toll pathway, but *Drosophila* containing Toll or Imd mutations are not killed after oral ingestion of bacteria [44]. Putative proteins with similarity to the Toll pathway components, cytokine-like Spaetzle (contig 34), Cactus (35), Tube (36), and Easter serine protease precursor (37), were identified in the present study. A contig with similarity to a *Drosophila* homolog of the TNF-α receptor, Wengen [45], was also identified (No. 38). Members of the TNFR superfamily mediate a wide spectrum of physiological and pathological events such as cell activation, proliferation, inflammation, and cell death.

The mitogen-activated protein kinase (MAPK) signaling cascade is involved in apoptosis and immune and stress responses. A MAPK was found to be putatively differentially expressed in the gut of *Lu. longipalpis* after infection with *Leishmania braziliensis* [9]. Contig 56 contains the ORF for a MAPK ortholog of p38 MAPK kinase (with a serine/threonine kinase domain). The p38 MAPK kinase of *D. melanogaster* is required for environmental stress response [46] and RNAi inhibition of the p38 ortholog in *Caenorhabditis elegans* showed that it functions as the downstream MAPK required for pathogen defense [47]. Numerous other putative kinases were detected in the dataset, including a CDK5 kinase ortholog (contig 57).

Another mechanism of host defense against pathogens involves the deliberate production of various kinds of reactive oxygen species (ROS). Homeostasis of redox balance in the gut of *Drosophila* is critical in regulating the interactions between the insect and its gut microbes [48]. Blood-feeding insects have a further consideration and must also neutralize the actions of free heme, which can generate ROS. Superoxide-based immune defenses are thought to be used in tsetse flies against trypanosomes by raising the levels of oxidative stress in the gut [49]. Blood-feeding insects such as tsetse flies possess a range of antioxidant enzyme systems such as superoxide dismutase (SOD), catalase, and peroxidases of various kinds [50].

Thioredoxin reductase (TrxR), rather than glutathione reductase, is the central enzyme regulator of redox balance characterized in *Anopheles* and *Drosophila.* Various elements of a TrxR antioxidant system were found in the current study. These include two sand fly contigs (Nos. 39 and 40) with similarity to different regions of the *Anopheles* TrxR 1 [51], others that are homologous with thioredoxin peroxidase (Nos. 41–44), as well as thioredoxin itself (No. 45). Also a putative copper–zinc SOD with homology to an *Anopheles* SOD was identified (46) and two contigs that appear to encode a peroxidase or catalase (47 and 48). *Pl. berghei* ookinete invasion of the midgut epithelium in *An. stephensi* induces peroxidases involved in tyrosine nitration, which leads to degeneration of the midgut cell [52], [53]. The “time bomb” theory suggests that the ookinetes must complete invasion of the midgut cell prior to generation of these toxic metabolites within the cell. These two contigs (Nos. 47 and 48) were most similar to one of five induced peroxidases (ENSANG00000019589). It is interesting that the same contigs were also found to possess similarity with *Drosophila* gut-expressed CG8913 immune-regulated catalase [48]. Another interesting potential antioxidant enzyme is xanthine dehydrogenase (XDH), the key enzyme in uric acid production. Uric acid is an antioxidant molecule and protects against hemin-induced oxidative stress, which is particularly relevant in blood-feeding insects [54]. A putative XDH with very high homology to *Anopheles* and *Drosophila* sequences (No. 49) was detected in the EST library.

The final group of potential immune genes identified are various enzymes/carrier proteins with housekeeping functions that have been implicated in antimicrobial defense in some way. The enzyme UDP-glucuronosyltransferase was deduced from another contig sequence (No. 50) and possessed very high similarity to the *An. gambiae* counterpart; this enzyme plays a central role in the detoxification and elimination of a wide range of endogenous and exogenous compounds and is specifically up-regulated by oral infection of protozoa (*O. muscaedomesticae*) but not bacteria in *Drosophila* [4]. Lysozymes have long been implicated in the gut defenses against microorganisms. A putative lysozyme containing a signal peptide and transmembrane component with homology to the i-type lysozymes [55] was identified (contig 51), with the Pfam search identifying a putative destabilase (Fig. S11).

### Digestive physiology proteins

Gut proteases/peptidases have been proposed to have various effects on *Leishmania* development and vice versa. Endo- and exopeptidases appear to be modulated during *Leishmania* growth in the digesting blood meal [56], [57], and it has been shown that disrupting the peritrophic matrix, thereby increasing the early exposure of parasites to midgut digestive enzymes during the transforming stages from amastigote to promastigote, reduces parasite survival [58]. *Leishmania* parasites will also be exposed to membrane-anchored exohydrolases, probably microvillar associated [59], throughout their development in the midgut. A large number of trypsin and chymotrypsin-like putative proteins were identified in the EST collection (representative sequences 58–63). Some of these are orthologs with proteases already studied in the sand fly *Ph. papatasi* [7], [8]. Three representative examples with predicted signal peptides (Nos. 58–60) were closely aligned with the *Ph. papatasi* trypsins 1–4 (Fig. S9). Three chymotrypsin-like sequences with signal peptides (contigs 61–63) aligned more closely to Ppchymo2 than to Ppchymo1 (Fig. S10).

**Supplementary Fig. S7:**
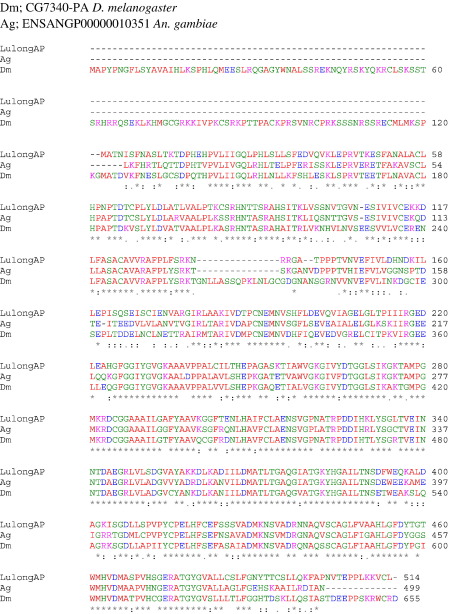
Clustal alignment of putative *Lu. longipalpis* leucine aminopeptidase.

**Supplementary Fig. S8:**
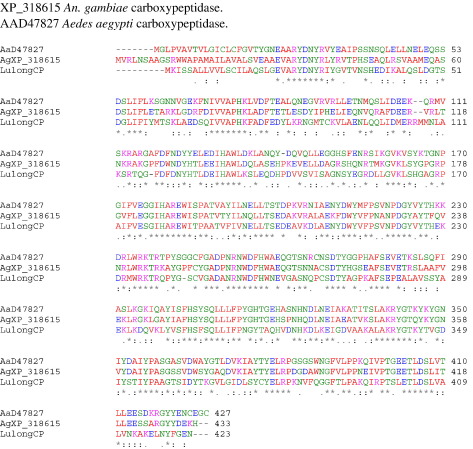
Putative Carboxypeptidase A sequence analysis comparison with *An gambiae* and *A aegypti* sequences.

**Supplementary Fig. S9:**
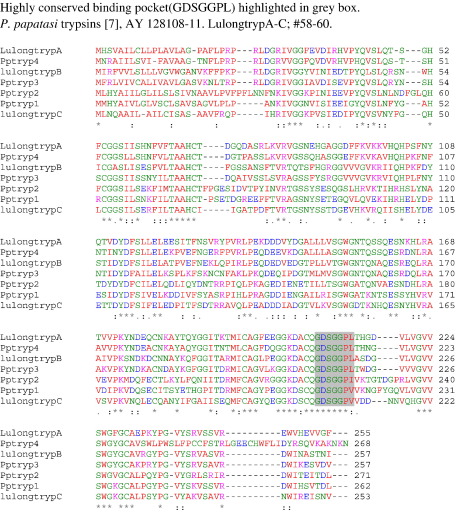
ClustalW alignment of putative *Lu. longipalpis* trypsins with *P. papatasi* midgut trypsins.

**Supplementary Fig. S10:**
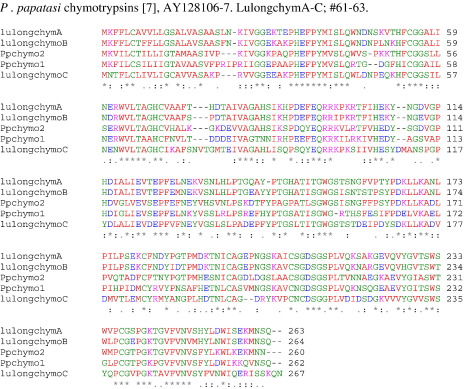
ClustalW alignment of putative *Lu. longipalpis* chymotrypsins with *P. papatasi* midgut chymotrypsins.

**Supplementary Fig. S11:**
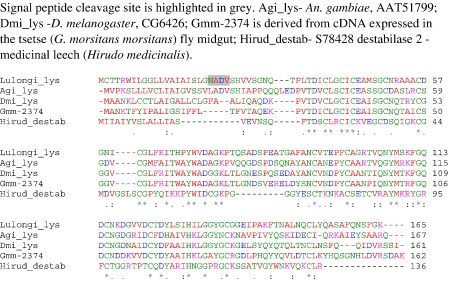
Putative Lysozyme I-type alignment.

A number of putative aminopeptidases (alanyl, glutamyl, leucyl) were identified in the EST database (contigs 64–68). Apart from digestive activities, aminopeptidases are involved in defense responses. Studies with *Aedes* and *Anopheles* suggest that aminopeptidase N induction is likely to be related to the mosquito response to *Plasmodium* in the midgut. Aminopeptidase is up-regulated in refractory *An. gambiae* strains [60] and significantly up-regulated 12 h following *Pl. gallinaceum* feeding to *Ae. aegypti* compared to uninfected insects [26]. Leucine aminopeptidase activities were significantly reduced in *Ph. papatasi* and *Ph. langeroni* following infection with *Le. major* [61], and a representative contig with homology to *An. gambiae* aminopeptidase is included here (Fig. S7). An ORF with a complete coding region for a putative secreted carboxypeptidase A with homology to a female gut-specific *Ae. aegypti* enzyme was also found (Fig. S8) [61].

Carbohydrase-digesting enzymes are important in digesting elements of both the blood and the sugar meal in sand flies. Sugars may also influence the anterior movement of *Leishmania* parasites through the gut after blood-meal digestion has finished [62] and glycosidases (contigs 70 and 71) may modify insect or parasite glycoproteins. Two putative amylases, one with homology to α-amylase (No. 72) and another to the amylase C-terminal β domain similar to midgut-expressed Agm2 (No. 73), were found [63]. Chitin is a polymer of the sugar *N*-acetyl galactosamine, and the importance of the PM as a barrier to development has been mentioned above. For these reasons both parasite and sand fly chitinases have been investigated [8], [64], [65], although other glycosidases may be at least as important in modifying the PM structure. Studies with mosquitoes and *Drosophila* indicate that chitinases exist in multigene families, with their deduced protein sequences having greatest homology to chitinase sequences from other species of insects [66]. The results from the current study suggest that this is also true for *Lu. longipalpis.* A midgut-expressed chitinase has been previously reported in *Lu. longipalpis* (AAN71763 [8]), but this has only 26% identity with contig 74, although the latter shares 78% identity with *Aedes* chitinase (T14075). Another possible chitinase is contig 75, which is 36% identical with AAN71763, but is more closely related to a so-called bacteria-responsive protein (BRP; AAS801380, 62% identity, Fig. S13 [67]). The contig 75 translated sequence lacks the highly conserved Glu residue at the active site, which is modified to a Gln in *Anopheles* and the putative *Lutzomyia* BRPs. *Anopheles* BRP1 and BRP2 are converted to smaller forms on exposure to bacteria and may have a role in the immune response, with the BRP2 protein expressed throughout the body, including the midgut.

**Supplementary Fig. S12:**
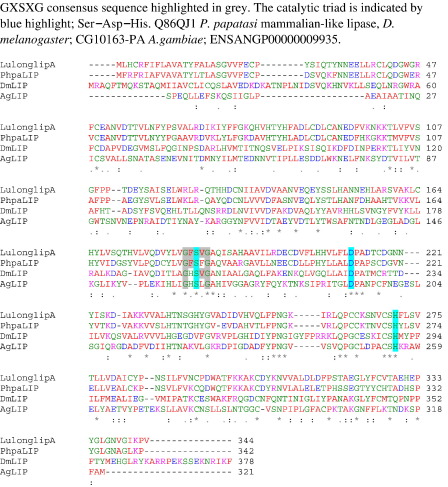
Alignment of putative Lipase.

**Supplementary Fig. S13:**
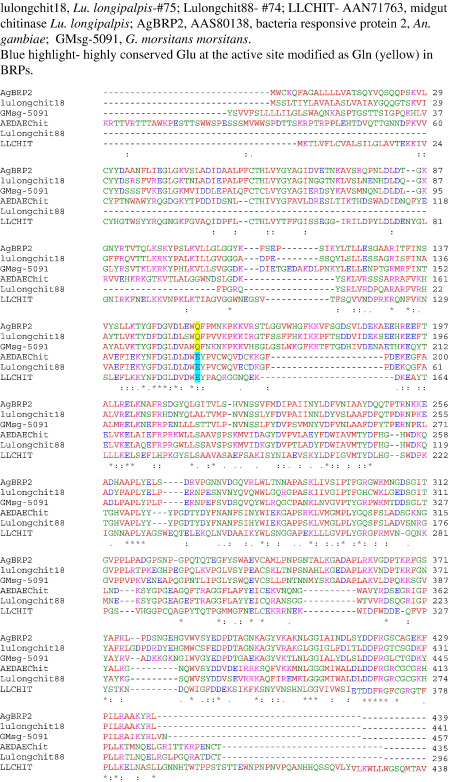
Alignment of putative chitinase-like sequences with homology to active and inactive–bacteria responsive proteins.

Other putative digestive enzymes of interest include a mammalian-like lipase previously characterized in *Ph. papatasi* [68]. The protein was expressed in the female accessory gland and gut, but not the thorax or head, indicating a role in lipid digestion. A complete ORF with signal peptide homologous with the *Ph. papatasi* lipase was found in the present study (contig 76; Fig. S12). Short-chain dehydrogenase/reductases possess a wide substrate spectrum, ranging from steroids, alcohols, sugars, and aromatic compounds to xenobiotics [69]. An *Aedes* SDR, which was expressed specifically in the adult gut, showed strong up-regulation after blood-meal digestion in a strain of mosquito permissive to parasite development [26]. Several putative SDRs were identified, two examples of which with homology to *Drosophila* and *Anopheles* counterparts are given in Table 1 (Nos. 77 and 78).

## Concluding remarks

Until recently the main focus of molecular studies on the sand fly–*Leishmania* relationship has been on the response of the parasite to the gut environment and the parasite-derived determinants of vector specificity. The impetus for this has been the publication/availability of the *Le. major* [70], *Le. Infantum,* and *Le. braziliensis* genomes (http://www.genedb.org) and the generation of a range of *Leishmania* mutants. These have provided us with many tools to investigate this part of the relationship but the response of the sand fly host to the parasite infection is still shrouded in mystery. The production of an EST library for an important vector of visceral leishmaniasis in South America now begins to redress this imbalance. This is an important resource in its own right, but is also part of an ongoing project to create a genome-wide cDNA microarray for *Lu. longipalpis* to investigate the insect component of the relationship on a global scale.

The EST project has identified a plethora of putative immunity genes. Historically interest in the immune mechanisms of insects has been focussed on the immune response to infections in the hemolymph, but attention is now directed increasingly toward epithelial responses. The insect immune response is known to be provoked by microbial growth in the gut lumen [4], [48], [71] and a sand fly defensin is induced during gut development of *Leishmania* in *Ph. duboscqi* [10]. Although the *Leishmania* parasite is confined to the gut lumen of the insect, there have been reports of parasites within the gut epithelial cells (R.J. Dillon, unpublished observations) and the point at which defensin is induced is currently unclear. The possibility that sand fly midgut epithelial GALEs are acting as PRRs in an immune sense is an interesting idea. Perhaps the binding of *Leishmania* promastigotes to microvillar GALEs activates the sand fly immune response, which serves to regulate microbial competitors of *Leishmania* in the gut lumen or the parasite population itself. The parasite burden in the gut can reach in excess of 50,000 gut^−1^ in *Lu. longipalpis,* but the insect succeeds in containing the parasite population and largely prevents their spread into the hemocoel. Unraveling the sand fly's response to the gut-confined *Leishmania* will contribute to our understanding of the nature and regulation of host gut–microbe interactions.

## Materials and methods

### Sand flies and Leishmania infections

The Jacobina strain of *Lu. longipalpis* (from Jacobina, Bahia, Brazil) kept at the Liverpool School of Tropical Medicine was used for this study. *Le. mexicana* (MNYC/BZ/62/M379) and *Le. infantum* (syn. *Leishmania chagasi*) (MHOM/BR/76/M4192) infections were maintained [15]. Female flies were infected by membrane feeding through a chick skin with a rabbit blood meal seeded with 2 × 10^6^ ml^− 1^ amastigotes of *Le. infantum* or *Le. mexicana* [15]. Samples of sand flies were checked for infection by dissection and microscopic examination of gut smears.

### Normalized cDNA library construction

Total RNA was isolated from whole bodies of 1950 female sand flies (approximately 1300 μg of total RNA). These included flies with the following range of physiological and infection histories: uninfected control flies fed on hamster blood or sugar meals (900 flies), infected flies harvested daily from day 1 to day 7 after the blood meal (*Le. mexicana,* 375 flies; *Le. infantum,* 125 flies), flies fed with the insect-derived bacterium *Pantoea agglomerans* and the insect pathogen *Serratia marcescens* (450 flies), and flies microinjected with *S. marcescens* (100 flies). Total RNA was isolated using the RNAqueous kit (Ambion) with the following modifications. Insects were immobilized by chilling on ice and placed in Ambion lysis buffer in lysing matrix tubes containing microbeads (BIO 101 Systems Lysing Matrix D tubes; Qbiogene) and lysed using a Fastprep instrument. The lysed suspension was immediately stored at −80°C until RNA purification using the RNAqueous protocol.

Messenger RNA was reverse transcribed using Superscript reverse transcriptase; double-stranded cDNA was synthesized with *Escherichia coli* DNA polymerase I and RNase H, size selected (> 350 bp) using Bio-Gel A-50-m columns (Bio-Rad), and directionally cloned in the pT7T3-Pac vector to generate a nonnormalized cDNA library as previously described [72]. It has been estimated that the prevalent and intermediate frequency classes of mRNA in a typical cell comprise as much as 50–65% of the total mRNA mass, but represent no more than 1000–2000 different mRNAs [72]. As a result, redundant identification of mRNAs of these two frequency classes would rapidly become overwhelming. Hence, a normalized library was derived from the nonnormalized library, so that the relative abundance of all clones fell within an acceptably narrow range. The normalized cDNA library was constructed according to method 4 of [72], except that Qiagen *Taq* DNA polymerase (Qiagen, Inc., CA, USA) was the polymerase used for PCR amplification of cDNA inserts to generate the driver population. A duplicate archive of all sequenced cDNA clones was established, one of a glycerol stock (an aliquot of the overnight culture used in template preparation) and the other a plasmid preparation (an aliquot of the template prepared above). cDNA clones will be made freely available to the research community on request.

### Sequencing and bioinformatics

About 15,000 colonies were randomly picked from the normalized library and templates prepared in 96-well format using Qiagen QIAwell 96 kits. Some additional clones were also sequenced from the unnormalized library. Templates were sequenced using a T3 or T7 primer with ABI Big Dye terminator kits. Sequences were assembled and edited using PHRED/PHRAP [19], with a minmatch score of 35, and all contigs and singlets imported into a gap4 database for viewing [73]. Depadded consensus sequences were exported for further analyses and automated annotation. Components of the annotation process included Blast analyses against several databases (e.g., UniProt, Ensembl *D. melanogaster*-specific and *An. gambiae*-specific databases, *Le. major* genome (v4.0), an in-house “all bacterial DNA sequences” database), followed by Interpro scans of the predicted *Lutzomyia* proteins to identify motifs/domains. GO terms were transitively annotated, based on similarity of predicted *Lutzomyia* proteins to GO-annotated *Drosophila* proteins. GO-annotated *Lutzomyia* proteins were further classified using a manually chosen GO-slim.

Analysis of sequences was performed using a range of software tools including the following: for detection of conserved function domains InterProScan (http://www.ebi.ac.uk/InterProScan/), for signal peptides Signal P 3.0 (http://www.cbs.dtu.dk/services/SignalP/), for N-linked glycans NetNGlyc 1.0 (http://www.cbs.dtu.dk/services/NetNGlyc/), for O-linked glycans NetOGlyc 3.1 (http://www.cbs.dtu.dk/services/NetOGlyc/), for transmembrane sequences TMHMM 2.0 (http://www.ch.embnet.org/software/TMPRED_form.html), and for GPI anchor sites big-PI Predictor (http://mendel.imp.ac.at/gpi/gpi_server.html).

### Data availability

All EST data have been deposited with EMBL and are available in the public databases (EMBL, GenBank). More information about the project and data availability can be obtained from http://www.sanger.ac.uk/Projects/L_longipalpis/.

## Figures and Tables

**Supplementary Fig. S14:**
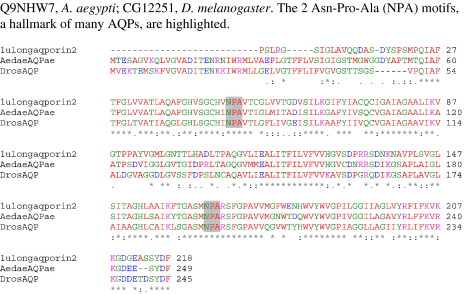
Putative aquaporin (Major Intrinsic Protein) in comparison with the *Drosophila* and *Aedes* orthologs.

## Appendix A Supplementary data

Supplementary Table 1 Matches with UniProt database and *Drosophila* and *Anopheles* proteins

Supplementary Table 2 BLASTN against Bacteria _DB database

Supplementary Table 3 Results of BLASTN against CDS of *L. major* with E < 10^−25^
