# Supplementary material for: Analysis of ESTs from Lutzomyia longipalpis sand flies and their contribution toward understanding the insect–parasite relationship
Source: Genomics. 2006 Dec;88(6):831–40. doi: 10.1016/j.ygeno.2006.06.011 (PMC2675706; doi:10.1016/j.ygeno.2006.06.011)
Supplement: Supplementary Table 1 — Matches with UniProt database and Drosophila and Anopheles proteins [file mmc1.doc]

Table S1 Matches with UniProt database and *Drosophila* and *Anopheles* proteins

Biological function

No hits Failed Similar % Novel Threshold (*E*=10^-5^ threshold)

Sand fly vs

UniProt 1624 2617 5962 41.6

*D.melanogaster* 1425 3145 5633 44.7

*An. gambiae* 1254 3432 5517 45.9

Total of 10203 sequences checked using BLASTX. UniProt release 5.7.
