# Supplementary material for: Analysis of ESTs from Lutzomyia longipalpis sand flies and their contribution toward understanding the insect–parasite relationship
Source: Genomics. 2006 Dec;88(6):831–40. doi: 10.1016/j.ygeno.2006.06.011 (PMC2675706; doi:10.1016/j.ygeno.2006.06.011)
Supplement: Supplementary Table 2 — BLASTN against Bacteria _DB database [file mmc2.doc]

Table S2 BLASTN against Bacteria _DB database

**Contig/Singlet Bacterial similarity Score e value**

NSFM-19h08.p1k NC_000913.2 *Escherichia coli* K12, 1699 0.0

NSFM-68h05.q1k NC_000913.2 *Escherichia coli* K12, 1604 0.0

NSFM-56d01.q1k NZ_AACJ01000095 *Haemophilus somnus*, 178 1e-42

NSFM-115f11.q1k NZ_AAHM01000324 *Burkholderia mallei* 174 4e-41

SFM-02a07.q1ka NZ_AACJ01000095 *Haemophilus somnus*, 170 7e-40

SFM-03e11.q1ka NZ_AAAP01003857 *Magnetospirillum magnetotacticum* 180 9e-43

SFM-02g12.p1ka NZ_AACJ01000093 *Haemophilus somnus*, 119 3e-24

NSFM-103b04.q1k NC_004337 *Shigella flexneri* 2a str., 1715 0.0

NSFM-99a03.q1k NZ_AAAN03000010 *Magnetococcus sp*., 121 4e-25

NSFM-68h05.p1k NC_004741 *Shigella flexneri* 2a str., 999 0.0
