# Supplementary material for: Analysis of ESTs from Lutzomyia longipalpis sand flies and their contribution toward understanding the insect–parasite relationship
Source: Genomics. 2006 Dec;88(6):831–40. doi: 10.1016/j.ygeno.2006.06.011 (PMC2675706; doi:10.1016/j.ygeno.2006.06.011)
Supplement: Supplementary Table 3 — Results of BLASTN against CDS of L. major with E < 10−25 [file mmc3.doc]

Table S3 Results of BLASTN against CDS of *L. major* with E < 10^-25^

**Contig/Singlet *Le. major* similarity Score e value**

NSFM-06a04.q1k LmjF35.1890 60S ribosomal protein L5, 26 0.0

NSFM-93h03.q1k LmjF33.0820 beta tubulin 801 0.0

NSFM-138e12.p1k LmjF32.1590 helicase-like protein 930 0.0

NSFM-56d01.q1k LmjF13.0390 alpha tubulin 121 4e-27

NSFM-149g01.p1k LmjF35.0420 40S ribosomal protein 1417 0.0

NSFM-50h06.p1k LmjF22.1470 hypothetical protein, 1118 0.0

NSFM-35e02.p1k LmjF36.3530 polyubiquitin, putative 583 1.0e-166

SFM-02a07.q1ka LmjF13.0390 alpha tubulin 260 1e-68

NSFM-101e07.q1k LmjF11.0970 40S ribosomal protein 714 0.0

NSFM-84g02.q1k LmjF02.0690 hypothetical protein, 143 4e-34

NSFM-57c12.q1k LmjF17.0550 RNA-binding protein, 129 9e-30

NSFM-84g02.p1k LmjF27.2610 hypothetical protein, 206 4e-53

NSFM-115h03.p1k LmjF28.2800 hypothetical protein, 1548 0.0

NSFM-87a04.q1k LmjF36.3530 polyubiquitin, 281 2e-75

NSFM-41c08.p1k LmjF22.1350 hypothetical protein, 406 1.0e-113

NSFM-42c01.q1k LmjF26.0280 hypothetical protein, 954 0.0

NSFM-46a03.p1k LmjF31.2460 lipase, putative 731 0.0

NSFM-15g06.p1k LmjF13.0390 alpha tubulin 141 1e-33

NSFM-162h01.p1k LmjF23.1480 hypothetical protein, 682 0.0

NSFM-165d08.p1k LmjF21.1566 hypothetical protein, 769 0.0

NSFM-67b06.p1k LmjF36.3530 polyubiquitin, 311 4e-84

SFM-01a07.q1k LmjF36.3530 polyubiquitin, 268 3e-71

NSFM-82a07.q1k LmjF32.1120 mitochondrial carrierprotein 529 1.0e-150

NSFM-02g09.p1ka LmjF33.0820 beta tubulin 139 2e-32

NSFM-33f09.q1k LmjF21.1070 40S ribosomal protein S23, 785 0.0
